# Virtual Simulation Versus Traditional Training for Orthodontic Bracket Positioning: A Pilot RCT

**DOI:** 10.1016/j.identj.2026.109530

**Published:** 2026-03-27

**Authors:** Danlin Lai, Baoliang Lai, Liang Xu, Xiaohong Huang

**Affiliations:** a Department of Stomatology, The First Affiliated Hospital, Fujian Medical University, Fuzhou, China; b Department of Stomatology, National Regional Medical Center, Binhai Campus of the First Affiliated Hospital, Fujian Medical University, Fuzhou, China; c Qingkou Town Health Center, Minhou County, Fuzhou, Fujian Province, China

**Keywords:** Orthodontic bonding, Virtual simulation, Dental education, Bracket placement accuracy

## Abstract

**Objective:**

The successful completion of orthodontic treatment largely depends on the accuracy of bracket placement. This study aimed to compare the impact of virtual simulation and traditional training on bonding, while also exploring student feedback regarding their learning journey.

**Methods:**

Twenty dental interns with no experience in orthodontics were randomly assigned either to a traditional training group (Group A) or a virtual simulation training group (Group B). Following their respective trainings, students placed orthodontic brackets on head-simulator models. Three-dimensional bracket deviations (mesiodistal, vertical, and buccolingual) from an ideal reference were quantified using the Geomagic Studio software. Participants’ motivation and satisfaction were also assessed with the ARCS (Attention, Relevance, Confidence, Satisfaction) motivation and learning experience satisfaction questionnaires. Due to the non-normal distribution of the data, the Mann-Whitney U test was used to compare differences between two groups.

**Results:**

The virtual simulation group had less deviation in bracket positioning in the mesiodistal and buccolingual directions than the traditional training group (*P* < .05). There was no statistically significant difference in the vertical directions (*P* > .05). Furthermore, the virtual simulation group scored higher in all questionnaire domains, although most of the differences were not statistically significant.

**Conclusion:**

Within the limitations of this pilot study, differences in selected aspects of the accuracy of bracket positioning were found to be associated with virtual simulation training. The findings suggest that virtual simulation provides learning outcomes comparable to those of traditional training without compromising technical accuracy from an educational perspective. Adoption of this technology is a great addition to modern dental educational curricula. However, more research with larger sample sizes is necessary. With the pilot nature of the study and limited statistical power, the results must be treated with caution.

## Introduction

Orthodontics, a subspecialty of stomatology, deals with the diagnosis, prevention, and treatment of malocclusions. The straight-wire technique, as described by Lawrence F. Andrews, remains the most widely used in clinical practice. Pre-adjusted brackets with built-in ideal tip, torque, angulation, and in-out position are used in this technique, which to a large degree negates archwire bending. Success in straight-wire orthodontics is thus, to a great extent, dependent on the accuracy of bracket bonding.^1^^,^^2^ Accurate placement minimizes chairside time, shortens overall treatment time, and is seen as a cornerstone of successful, efficient, and esthetic treatment outcomes.^3^

The traditional methods of bracket placement have been enhanced by standardized clinical tools such as bracket placement guides,^4^^,^^5^ location of the facial axis (FA) point,^6^ and reference charts,^7^ for prescription-specific bracket placement. These methods, when properly used, improve the accuracy of positioning and are essential parts of modern orthodontic practice.

For the purpose of imparting such standards to young clinicians, bracket placement training is conventionally done on plaster models. Students learn to measure and mark the long axis of the clinical crown and place brackets with sticky wax. Instructors then give subjective, visual feedback for correction. Students carry over this method to clinical internships, where they observe and then do the procedure under supervision.

However, despite the fact that such training protocols have been established, the placement of brackets is a technique-sensitive procedure, especially in the initial stages. Even though the operator variation is kept to a minimum in the laboratory environment, the process of bracket placement involves the accurate identification of certain landmarks and the visual dexterity of the operator. Variability among inexperienced learners may thus lead to clinically significant differences in positioning.^8^

The current study does not question the validity of traditional methods. Instead, it focuses on the educational issue of how to ensure consistent skill training in the early learning stages. Virtual simulation technology may provide a complementary tool with the potential for creating standardized, repeatable, and feedback-driven learning environments.^9^ By using computer-generated three-dimensional(3D) simulations, learners can practice clinical tasks in a controlled and immersive environment.

This study thus quantitatively assessed the accuracy of bracket positioning between interns trained with traditional teaching methods and virtual simulation training. Perceived teaching effectiveness and learning experience were also assessed using structured questionnaires. The results are intended to contribute to the development of orthodontic education and to investigate the potential role of virtual simulation in facilitating high-quality clinical training.

## Materials and methods

### Patient and model preparation

**Fig. 1:**
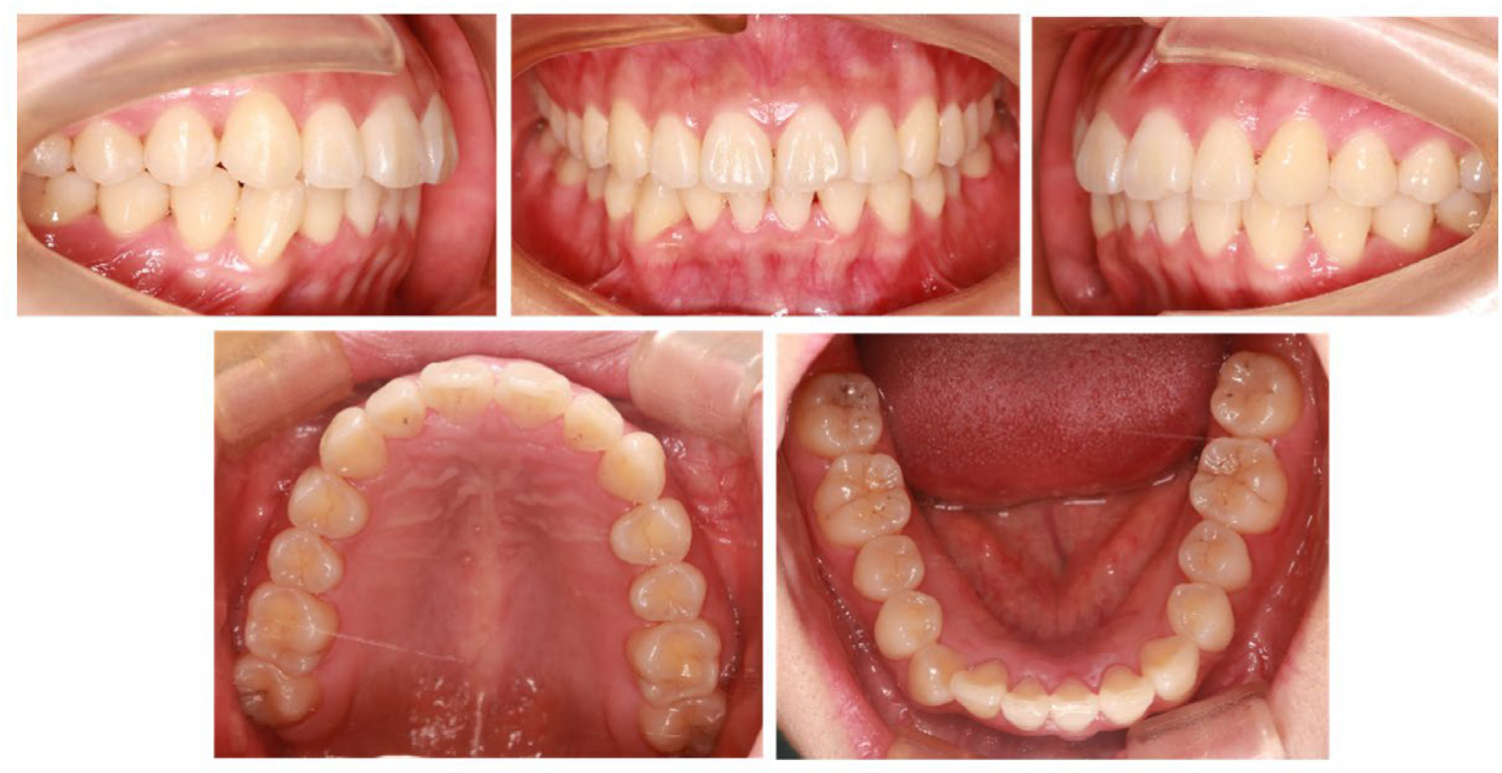
Intraoral photographs of the patient.

A female orthodontic patient from the Department of Orthodontics of the First Affiliated Hospital of Fujian Medical University was selected for this study. The patient presented with a chief complaint of tooth misalignment, and clinical exam revealed bilateral Class I molar relationships with mild crowding in the mandible. The intraoral photographs of the patient are illustrated in Fig. 1.

Digital dental impressions were acquired at an accuracy of 20 µm through the use of an intraoral scanner (3Shape Trios, Denmark).^10^ This was transformed into a 1:1 virtual three-dimensional digital dental model. The model data were then exported in STL(Standard Tessellation Language)format and used to print a series of standardized bonding simulation models using a 3D printer (3D Systems, USA). These models served as the standardized training materials for all participants.

### Study participants and grouping

20 dental interns at the First Affiliated Hospital of Fujian Medical University who had never received formal training in orthodontic bracket placement were recruited as volunteers between May and June 2025. A total of 20 Participants were randomly assigned to either the Traditional Training Group (Group A) or the Virtual Simulation Training Group (Group B). Both groups had a total of 10 Participants. In order to ensure the integrity of the study, the allocation sequence was generated by an independent researcher who was not connected with the training or the outcome assessment. A computerized random number generator was employed to generate the allocation sequence. The use of opaque, sealed, sequentially numbered envelopes ensured allocation concealment. The envelopes were not opened until the participant had been enrolled. Baseline demographic information, including age and gender, was statistically similar in the two groups (*P* > .05), enabling balanced comparison.

The ethical principles outlined in the Declaration of Helsinki guided the conduct of this research throughout its duration. The study is an educational research study, which entails only the introduction of new digital teaching methods as an addition within the orthodontic practice of interns. Anonymized intraoral scan data and photographs of patients were used. It will have no effect on the overall teaching process and quality, and no disagreement regarding the fairness of teaching. This research was approved by the First Affiliated Hospital of Fujian Medical University Ethics Committee (Decision No: MTCA, ECFAH of FMU |2015|084-2) after which it was conducted. Written informed and oral consent were obtained from the study participants. This was an exploratory pilot randomized controlled educational study and was not registered in a clinical trial registry.

### Training protocol and bracket bonding

To standardize the teaching level and reduce variability between instructors, both groups were taught by the same senior orthodontist. A standardized teaching process was followed. Theoretical teaching was conducted in the same manner with the same teaching materials. Basic principles such as FA point location and bracket placement criteria were taught in the same way. The time allocated for the lectures and the practical sessions was the same. All students initially underwent a didactic lecture on bracket bonding using the Andrews’ clinical crown center technique, after which they received their respective training modality:

Group A (Traditional): Conducted bracket positioning practice on the 3D-printed physical models with adhesive wax.

Group B (Virtual Simulation): Received virtual bracket positioning training through the assistance of Ortho Analyzer software (3Shape Dental Systems, Denmark), providing a multi-angle, rotatable digital simulation system.

**Fig. 2:**
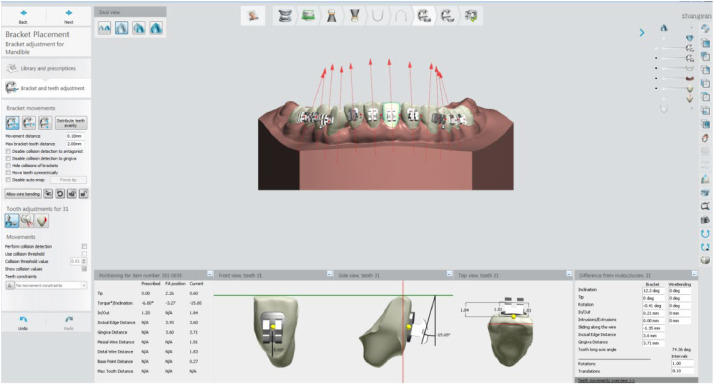
Virtual simulation training interface of Ortho Analyzer.

**Fig. 3:**
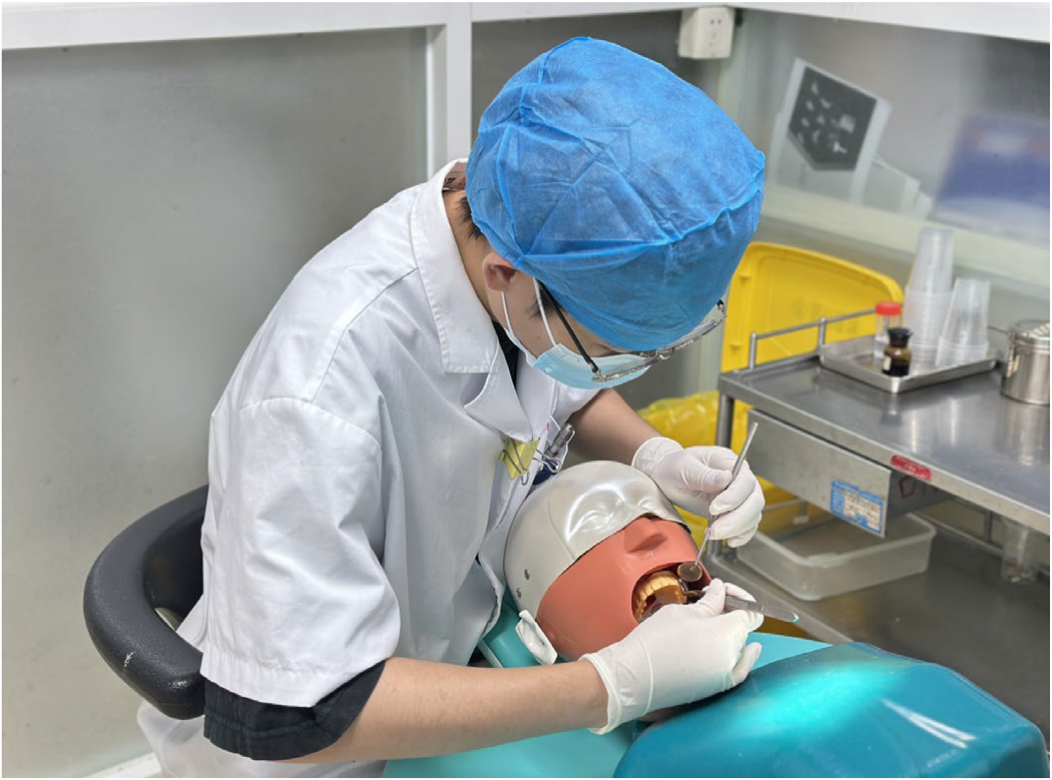
Dental models mounted on the head simulator, replicating clinical posture and working environment.

All brackets employed were 0.022 × 0.028-inch MBT brackets (Shinye Orthodontic Products, Hangzhou, China). Fig. 2 illustrates the training interface of Ortho Analyzer. After training, both groups mounted their 3D-printed models on head-simulators (NISSIN, NT1CM-2000, Japan) to simulate real clinical posture and working conditions (Fig. 3). To make the results of the experiment comparable and to eliminate bias related to the material, both groups worked with the same dental models made using 3D-printed models. This made it possible to compare only the type of training.

In order to achieve high internal validity and reduce anatomical differences, a single standardized dental model was used for this experiment. Students then bonded brackets onto all maxillary and mandibular anterior teeth and premolars (FDI: 11-15, 21-25, 31-35, 41-45). The molar region was excluded at this stage due to greater procedural complexity and limited visibility, with the possibility of introducing additional technical variability among inexperienced dental interns. After bonding, the models were removed and scanned with a 3Shape Trios to achieve digital data for assessment.

As the ideal reference, two experienced orthodontic teachers bonded brackets on the same model separately with a single clinical crown center method. Instead of using digital methodologies, these ideal reference points were manually defined by the specialists to ensure a clinically realistic manner. They were also scanned, and the digital information thus obtained was used as the reference standard for further deviation analysis.

### Assessment of bracket positioning accuracy

**Fig. 4:**
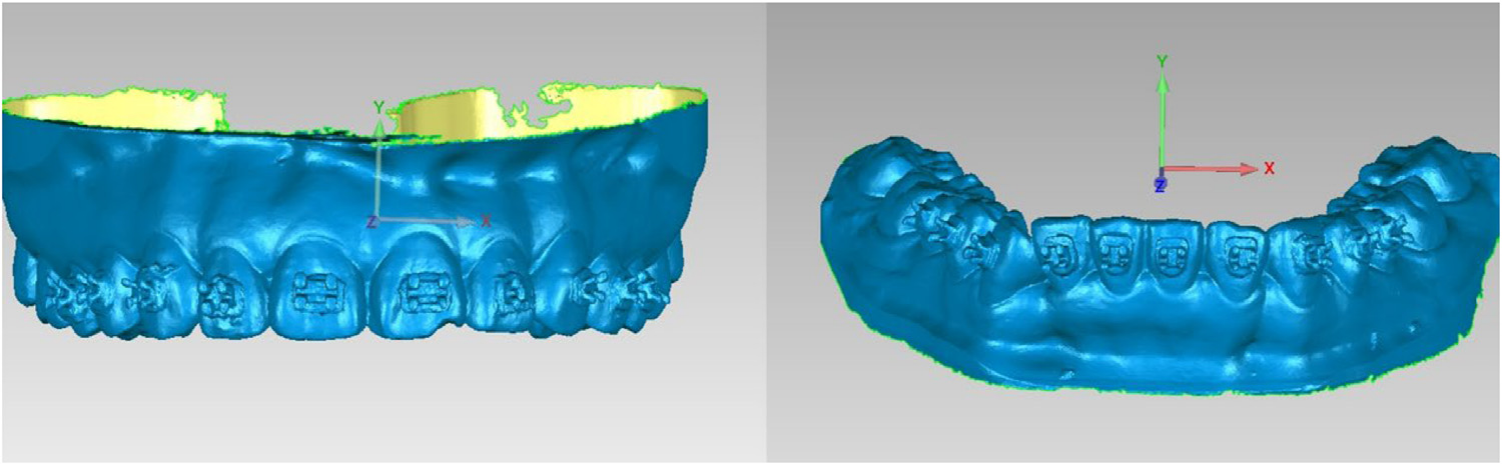
A and 4B Maxillary and mandibular coordinate system.

**Fig. 5:**
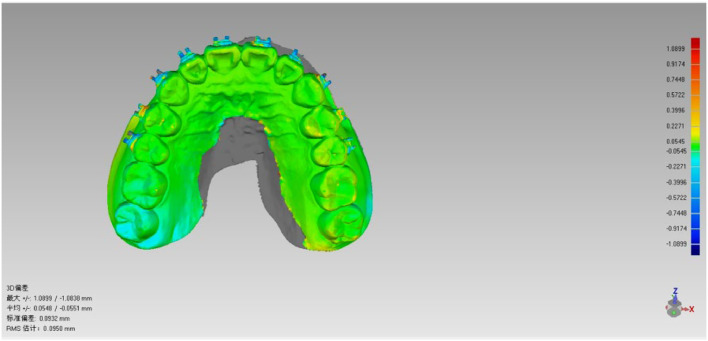
Maxillary global deviation analysis performed through automated best-fit superimposition.

Scanned STL files of the reference standard, Group A, and Group B, were imported into Geomagic Studio 2014 (3D Systems, USA). The reference standard model was defined as the baseline coordinate system (Fig. 4a and b). The Group A and Group B datasets were registered to this baseline through best-fit registration, with global registration deviation restricted to <0.1 mm (Fig. 5).

**Fig. 6:**
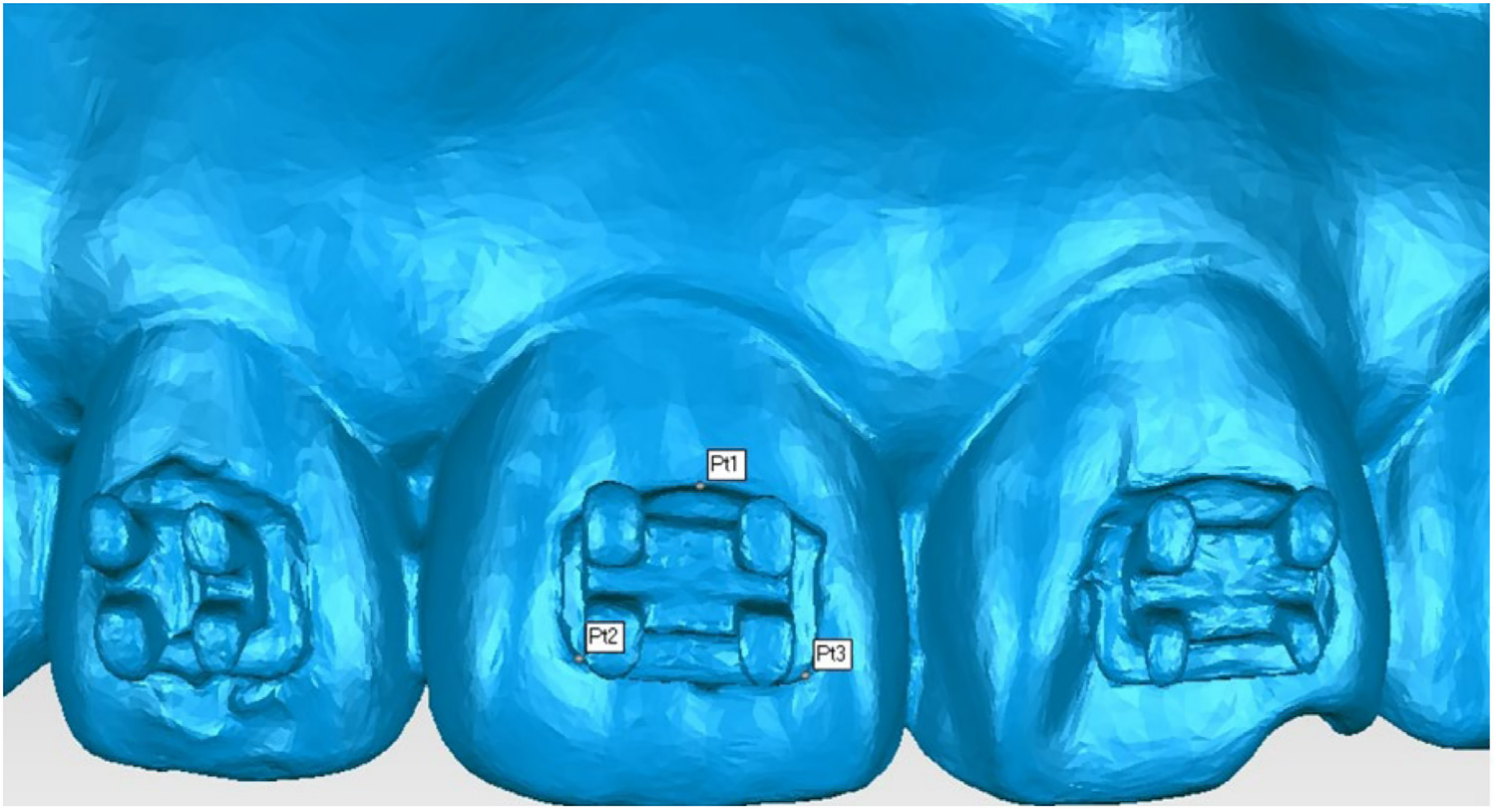
Reference points for bracket measurement. Pt1,Gingival Point; Pt2,Distal Point; Pt3,Mesial Point.

To quantify bracket position, three stable reference points were defined on each bracket surface: a gingival point (Pt1, Point Target 1), a distal point on the incisal edge (Pt2, Point Target 2), and a mesial point on the incisal edge (Pt3, Point Target 3). These points defined a stable local coordinate system for each bracket (Fig. 6). By comparing the spatial orientations of these local systems, the 3D positional deviations of the bonded brackets were quantified in the mesiodistal (X-axis), vertical (Y-axis), and buccolingual (Z-axis) directions. The absolute values of these deviation measurements were used for analysis.

To reduce the risk of observer bias, a blinded assessment protocol was implemented. All digital models were de-identified and randomly numerically coded by an independent third party, ensuring the assessor remained unaware of group allocations (Traditional vs Virtual Simulation) until data analysis were complete.

All the measurements were carried out independently by two calibrated examiners using a standardized digital measurement procedure. Before the study, both examiners were trained for calibration to attain consistency in reference points identification and measurement procedures. In case of any discrepancy, the measurements were reviewed jointly, and a consensus value was decided for final analysis.

### Questionnaire surveys

Learning motivation and satisfaction of students were measured by two validated questionnaires: the ARCS (Attention, Relevance, Confidence, Satisfaction) Learning Motivation Questionnaire^11^ and a Learning Experience Satisfaction Questionnaire.

### Statistical analysis

Data processing and statistical evaluations were carried out using SPSS software (version 27.0; IBM Corp).

### Bracket positioning deviation analysis

Group A and Group B bracket position deviation values were compared among themselves and also with the reference standard. Because the result of the Shapiro–Wilk test showed the distribution of the discrepancies of position was not normally distributed, we used the Mann-Whitney U test, for the comparison of inter-group accuracy. To ensure a proper interpretation of the result due to the distribution, the result was expressed using the Median [Q1, Q3]. The level of significance was set as *P* < .05. 1200 measurements (400 brackets, 3 dimensions each) were analyzed to calculate deviations in the mesiodistal, buccolingual, and vertical directions.

Because of the exploratory nature and limited sample size of this pilot study, the data from the maxillary and mandibular arches were combined for a general assessment of accuracy rather than being analyzed by strata. Likewise, more complex statistical models for clustering effects were not used. A post-hoc power analysis was conducted using the G*Power 3.1 software, with a focus on the major outcome measures in relation to the X, Y, and Z axes, despite the sample size not being determined prior to the study.

### Questionnaire data analysis

**Fig. 7:**
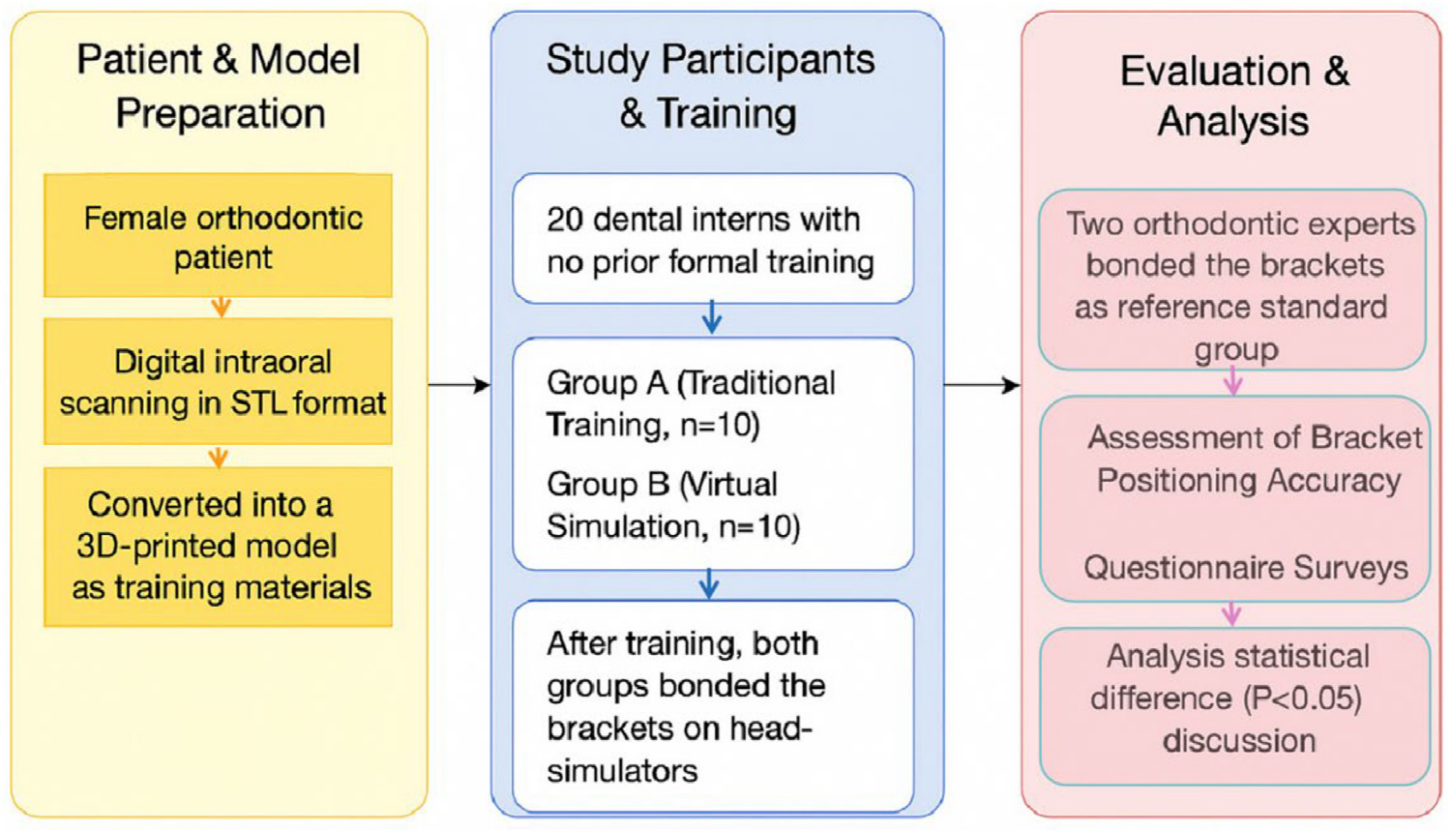
Flow chart of the research.

Both questionnaires had closed-ended items on a 5-point Likert scale. Each questionnaire score was analyzed as continuous data. The ARCS questionnaire was scored according to its four pre-determined dimensions: Attention, Relevance, Confidence, and Satisfaction. The Learning Experience Satisfaction Questionnaire was scored according to three dimensions: Content and Design, Operation and Tools, and Learning Outcomes. The median score of each dimension of the two instruments was calculated and compared between the two groups by the Mann–Whitney U test in order to identify the differential effects of the training methods on learning motivation and satisfaction. The specific experimental process is shown in Fig. 7. The CONSORT 2025 statement for randomized controlled trials was followed in the conduct and reporting of this study.

## Results

**Fig 8:**
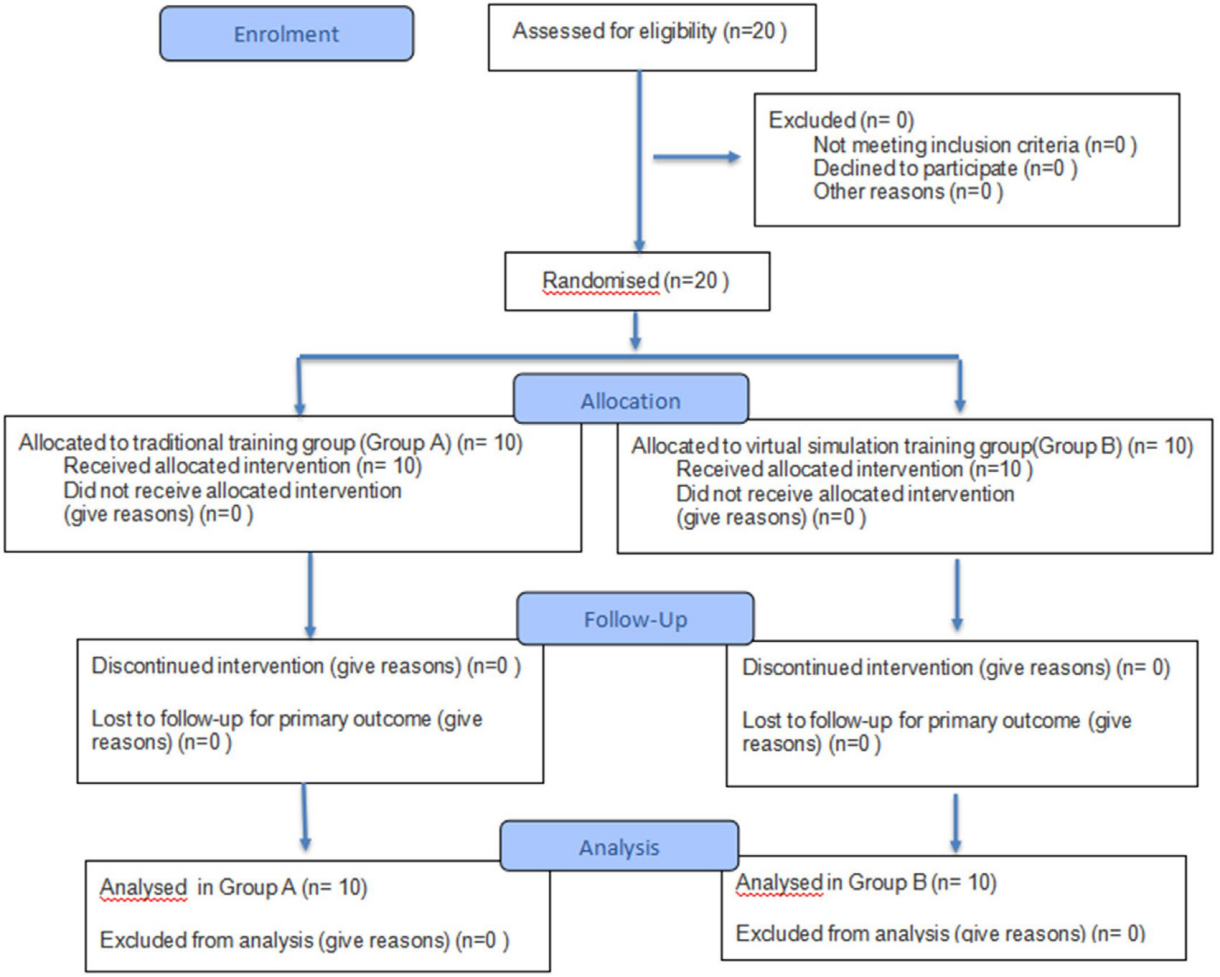
CONSORT 2025 flow diagram for the pilot study.

A total of 20 dental interns were screened for eligibility, and all of them were found to be eligible for the study. The participants were then randomly assigned to either the traditional training group or the virtual simulation group. There were no losses to follow-up or exclusion from analysis for the participants. The flow of the participants is shown in Fig. 8.

### Bracket positioning accuracy

The accuracy of the bracket positioning was assessed by measurement of median and interquartile range (Q1, Q3) of mesiodistal (X-axis), vertical (Y-axis), and buccolingual (Z-axis) deviations in both groups. The post-hoc power analysis indicated that the statistical power for the measurements along the X, Y, and Z axes was 0.091, 0.318, and 0.675, respectively. Results for different tooth positions are shown in Table 1, Table 2, Table 3.

**Table 1:** Mesiodistal (X-axis) deviations: median and interquartile range.

		Group A	Group B	*P*
		X/Mesiodistal (mm)	X/Mesiodistal (mm)	
Tooth Type	*n*	Median (Q1, Q3)	Median (Q1, Q3)	
Incisor	80	0.233 (0.117, 0.404)	0.174 (0.094, 0.332)	<.05
Canine	40	0.230 (0.089, 0.433)	0.255 (0.138, 0.490)	>.05
Premolar	80	0.301 (0.144, 0.547)	0.269 (0.123, 0.497)	>.05
Total	200	0.250 (0.121, 0.460)	0.219 (0.111, 0.415)	=.05

**Table 2:** Vertical (Y-axis) deviations: median and interquartile range.

		Group A	Group B	*P*
		Y/Vertical (mm)	Y/Vertical (mm)	
Tooth Type	*n*	Median (Q1, Q3)	Median (Q1, Q3)	
Incisor	80	0.256 (0.114, 0.388)	0.215 (0.111, 0.374)	>.05
Canine	40	0.207 (0.110, 0.404)	0.254 (0.127, 0.428)	>.05
Premolar	80	0.353 (0.167, 0.537)	0.323 (0.154, 0.492)	>.05
Total	200	0.273 (0.125, 0.468)	0.265 (0.123, 0.433)	>.05

**Table 3:** Buccolingual (Z-axis) deviations: median and interquartile range.

		Group A	Group B	*P*
		Z/Buccolingual (mm)	Z/Buccolingual (mm)	
Tooth Type	*n*	Median (Q1, Q3)	Median (Q1, Q3)	
Incisor	80	0.263 (0.132, 0.470)	0.177 (0.079, 0.308)	<.001
Canine	40	0.285 (0.116, 0.536)	0.277 (0.151, 0.553)	>.05
Premolar	80	0.367 (0.152, 0.632)	0.390 (0.178, 0.654)	>.05
Total	200	0.312 (0.136, 0.452)	0.245 (0.113, 0.501)	<.05

### Mesiodistal (X-axis) position deviation

In regard to incisor placement, the virtual simulation group (Group B) showed a significant reduction in mesiodistal deviation compared with the traditional training group (Group A). The values were 0.174 mm for Group B and 0.233 mm for Group A (*P* < .05). Apart from that, the group under virtual simulation also experienced a more restricted data distribution, as indicated by its narrower interquartile range (0.094, 0.332) compared to the traditional training group (0.117, 0.404), indicating lower variability in this region. Although Group B also recorded slightly lower median deviations in the canine and premolar areas, these differences did not reach statistical significance (*P* > .05). In overall median deviation, Group B had a lower value than Group A, but this difference was not statistically significant (*P* = .05).

### Vertical (Y-axis) position deviation

Group A had higher median values of vertical deviation than Group B at all tooth positions. The differences, however, were not significant (*P* > .05).

### Buccolingual (Z-axis) position deviation

In the incisor region, the median buccolingual deviation of Group B (virtual simulation group) was less than that of Group A (traditional training group) [0.177 mm (0.079, 0.308) vs 0.263 mm (0.132, 0.470); *P* < .001], with the reduction approximately 32.69%. The overall median deviation of Group B was also less (0.245 mm vs 0.312 mm; *P* < .05). Because the pilot nature and small sample size of the current study are considered, the results are presented descriptively and may indicate a possible advantage in the accuracy of buccolingual positioning for the virtual simulation group.

### Questionnaire results

Results of ARCS learning motivation and learning experience satisfaction questionnaires are displayed in Table 4, Table 5, respectively.

**Table 4:** Comparison of ARCS Learning Motivation scale scores between two groups.

Dimension	Group A Median	Group B Median	*P*
Attention	5	5	>.05
Relevance	5	5	>.05
Confidence	5	5	=.05
Satisfaction	4.75	5	>.05
Total	4.8	5	>.05

**Table 5:** Comparison of Learning Experience Satisfaction scale scores between two groups.

Dimension	Group A Median	Group B Median	*P*
Content and Design	4.83	5	>.05
Operation and Tools	4.67	5	=.05
Learning Outcomes	5	5	>.05
Total	4.75	5	>.05

### ARCS learning motivation

Group B also recorded higher median values for all four ARCS dimensions (Attention, Relevance, Confidence, and Satisfaction) compared to Group A. However, the results showed borderline significance (*P* = .05) for the Confidence dimension. There were no statistically significant differences (*P* > .05) for the remaining dimensions.

### Learning experience satisfaction

Group B scored higher medians on all three dimensions of the satisfaction questionnaire. Regarding the Operation and Tools dimension, the *P* value was .05. For the Content and Design and Learning Outcomes dimensions, there were no statistically significant differences (*P* > .05).

## Discussion

Statistically significant differences were found in mesiodistal and buccolingual positioning of the brackets, especially in the incisor area, where the virtual simulation group showed smaller median deviations. No statistically significant differences were found in the vertical dimension. These results indicate a possible advantage of virtual simulation in specific positioning parameters, but due to the small sample size, the results should be interpreted with caution. Our findings agree with those of earlier research by Tang and Oliveira,^12^, ^13^, ^14^ which showed improvements in orthodontic training performance using virtual simulation systems. The potential benefit of virtual simulation is not in replacing traditional training, but in providing a structured, repeatable, and risk-free environment that could potentially aid in the early development of skills.^15^^,^^16^ The absence of a statistically significant difference in vertical positioning, despite the trend for improvement in the virtual simulation group, is an interesting finding. The finding is in agreement with Xue and Aboujaoude’s study,^17^^,^^18^ which indicated that traditional training has not improved the accuracy in the vertical direction. Our finding, however, is different from other studies^3^ that demonstrated a significant vertical benefit with virtual simulation. This may be due to methodological differences, including sample size, tooth selection, and statistical power, which can affect the statistical power and generalizability of findings.

Besides technical competence, this research also demonstrates the pedagogical merit of virtual simulation. The questionnaire results showed a higher median score in the virtual simulation group for a number of motivational and satisfaction domains. Although most of the differences were not statistically significant, the Confidence dimension approached statistical significance (*P* = .05). The findings may indicate a potential association with learner self-efficacy, which is consistent, and it favors virtual simulation, which could be due to increased learner engagement and self-confidence. The interactive and repeatable nature of virtual simulation may also facilitate the provision of instant feedback and enable students to practice without risk.

Though the results should be viewed with caution due to the small sample size used in the study, there is a clear upward trend in the results across the different domains. Similar results have also been observed by De Boer,^19^ in which three-dimensional visualization in virtual worlds was shown to improve both the performance and enjoyment of learners in the learning process. A combination of virtual simulation with traditional methods may also be viewed as a balanced approach.^20^, ^21^

Multiple comparison corrections are not employed, as this is a pilot study of an exploratory nature. Thus, the *P*-values should be considered with caution, as there may be an increased risk of Type I error. In addition, because multiple brackets were placed by the same participant, there may be clustering (pseudoreplication) bias in the analysis, and the results may be overstated in terms of significance. These results will be presented as preliminary findings to inform future research.

This study is innovative in two key aspects. First, compared to previous studies that have particularly considered general accuracy or one-dimensional outcomes, our research quantifies the virtual simulation performance for each location of the three-dimensional tooth region. Second, it goes beyond technical measurement by adding questionnaires to gauge student motivation and learning experience. Although the use of a single standardized model enhanced experimental control, it could be a limitation if the results are extended to allow a wider range of more complex scenarios. Future research with larger sample sizes, stratified analysis, and the use of multilevel statistical modeling will be necessary to replicate these preliminary findings and to assess the generalizability of training effects to clinical practice.

## Conclusion

The pilot study has shown that virtual simulation training significantly reduced median deviations for mesiodistal and buccolingual positions of the brackets, especially in the incisor area. No statistically significant difference was observed in the vertical dimension.

Regarding educational outcomes, the virtual simulation group showed a higher median in several dimensions related to motivation and satisfaction, with the Confidence dimension showing a *P* value of .05. This indicates a potential benefit from virtual simulation in terms of educational outcomes.

By combining objective performance data with educational outcome measures, this study offers preliminary evidence of the possible utility of virtual simulation in orthodontic education. From an educational perspective, virtual simulation provides learning outcomes comparable to those of traditional training without compromising technical accuracy. However, in view of the small sample size, the exploratory nature of the study, the results obtained should be viewed cautiously, and further research is needed to confirm the findings.

### Limitations and future directions

Despite these strengths, our study has a few known limitations that give way to future research. First, while numerous measurements were taken, all brackets were placed on a single, standardized model. Future studies must involve a greater range of clinical models, including varying arch forms and dental anomalies, in order to enhance the generalizability of our findings. Second, the anterior teeth and premolars, but not the molar area, were evaluated in this investigation, and the molar area must be included in future studies. Furthermore, our investigation compared the end-result accuracy of the two training approaches without breaking down the precise procedural mechanisms driving the observed differences. Future investigations can employ advanced techniques such as motion capture technology to track operator movements in real time so that the ultimate causes of positioning errors can be teased apart.

From these limitations, some directions of interesting research emerge:

### Sample size and study design

This study was carried out as an exploratory pilot study without calculating the prospective sample size. Although the post hoc power calculation outcome indicates a restricted ability to detect small to moderate effects, the post hoc power calculation outcome, in no way, can be considered a substitute for the lack of prospective power calculation. Thus, the findings must be replicated in appropriately powered multi-center randomized controlled trials with a larger and more diverse population.

### Methodological and statistical considerations

There are a few statistical limitations that need to be considered. First, although the measurements were carried out by two calibrated examiners, there was no formal statistical evaluation of the data to verify inter-rater reliability. In future studies, repeated measurements will be taken to assess inte-rater reliability to ensure more valid results.

Secondly, there was no correction for multiple comparisons, and this might have led to a possible Type 1 error. The study was an exploratory pilot study, and hence, analysis was done with the aim of determining initial patterns.

Thirdly, there could have been clustering (pseudoreplication) due to the fact that the multiple brackets of the same participant were considered to be independent data. This could have had an effect on the variance and statistical significance. Future studies should use multilevel or mixed models to control for clustering at the participant level.

In addition, while non-parametric tests were appropriately applied, more advanced statistical modeling should be considered in larger studies to improve analytical robustness.

### Scope of tooth and arch analysis

The molar area was not included in the study to make the task uniform for inexperienced clinicians. Nevertheless, the positioning of molar brackets is of clinical interest. Future studies should include posterior teeth and more complex malocclusion models to allow for the assessment of three-dimensional accuracy in full arches.

Moreover, the maxillary and mandibular teeth were considered together in the current study. However, future studies should perform stratified analyses to assess the potential arch-specific effects.

#### Learning Efficiency

Although both groups were provided standardized instructional time, individual self-directed practice time was not quantitatively assessed. Differences in ‘time on task’ could have affected results. Future research should include an objective learning efficiency assessment.

#### Transferability and long-term follow-up

However, the current study did not assess the transfer of skills to actual clinical environments or retention of skills. Future studies should include follow-up to assess the long-term educational effects of virtual simulation on clinical transferability.

#### Economic Evaluation

Formal cost-effectiveness analysis was not possible in the context of the pilot study. Virtual simulation systems provide great advantages with regard to scalability and potential to facilitate repetitive high-fidelity training without repeated use of physical resources, although it demands an initial investment of funds in software and hardware. Comprehensive economic analysis studies should be included in future large-scale studies to assess the feasibility and return on investment in educational settings in a variety of academic contexts.

#### Future Technological Development

New digital technologies, such as artificial intelligence and virtual reality, could potentially improve accuracy and workflow integration in orthodontic education. Future studies should investigate the potential use of these technologies within sound experimental designs to facilitate accurate, reliable, and clinically valid orthodontic educational platforms.^22^^,^^23^

## Funding

This study was supported by the Young and Middle-Aged Teachers Education Research Project of Fujian Province (Grant No. JAT232010) and the Medical Education Research Project of the 10.13039/501100006765Chinese Medical Association Medical Education Branch (Grant No. 2023B062).

## CRediT authorship contribution statement

**Danlin Lai:** Conceptualization, Methodology, Formal analysis, Writing – original draft, Project administration. **Baoliang Lai:** Conceptualization, Resources, Supervision, Data curation. **Liang Xu:** Investigation, Methodology. **Xiaohong Huang:** Conceptualization, Methodology, Investigation, Writing – review & editing.

## Conflict of interest

The authors declared no potential conflicts of interest with respect to the research, authorship, and/or publication of this article.

## Appendix Supplementary materials

Image, application 1
