# Supplementary material for: Virtual Simulation Versus Traditional Training for Orthodontic Bracket Positioning: A Pilot RCT
Source: Int Dent J. 2026 Mar 27;76(3):109530. doi: 10.1016/j.identj.2026.109530 (PMC13058975; doi:10.1016/j.identj.2026.109530)
Supplement: Supplementary file 1 [file mmc1.docx]

|  | Section/topic | No | CONSORT checklist item description | Reported on page no. |
| --- | --- | --- | --- | --- |
|  | **Title and abstract** | | |  |
|  | Title and structured abstract | 1a | Identification as a randomised trial | Page 1 |
|  |  | 1b | Structured summary of the trial design, methods, results, and conclusions | Page 1-2 |
|  | **Introduction** | | |  |
|  | Background and rationale | 2a | Scientific background and rationale | Page 2-3 |
|  | Objectives | 2b | Specific objectives related to benefits and harms | Page 2-3 |
|  | **Methods** | | |  |
|  | Trial design | 3a | Description of trial design including type of trial (eg, parallel group, crossover), allocation ratio, and framework (eg, superiority, equivalence, non-inferiority, exploratory) | Page 5 |
|  | Changes to trial protocol | 3b | Important changes to the trial after it commenced including any outcomes or analyses that were not prespecified, with reason | None |
|  | Trial setting | 4a | Settings (eg, community, hospital) and locations (eg, countries, sites) where the trial was conducted | hospital |
|  | Eligibility criteria | 5a | Eligibility criteria for participants | Page 5 |
|  |  | 5b | If applicable, eligibility criteria for sites and for individuals delivering the interventions (eg, surgeons, physiotherapists) | Page 5 |
|  | Intervention and comparator | 6 | Intervention and comparator with sufficient details to allow replication. If relevant, where additional materials describing the intervention and comparator (eg, intervention manual) can be accessed | Page 5-6 |
|  | Outcomes | 7a | Prespecified primary and secondary outcomes, including the specific measurement variable (eg, systolic blood pressure), analysis metric (eg, change from baseline, final value, time to event), method of aggregation (eg, median, proportion), and time point for each outcome | Page 9-10 |
|  |  | 7b | Changes to outcomes | None |
|  | Sample size | 8a | How sample size was determined, including all assumptions supporting the sample size calculation | NA Page 20  (exploratory study) |
|  |  | 8b | Explanation of any interim analyses and stopping guidelines | NA Page 20  (exploratory study) |
|  | Randomisation: |  |  |  |
|  | Sequence generation | 9a | Who generated the random allocation sequence and the method used | Page 5 |
|  |  | 9b | Type of randomisation and details of any restriction (eg, stratification, blocking and block size) | Page 5 |
|  |  |  |  |  |
|  | Allocation concealment mechanism | 10 | Mechanism used to implement the random allocation sequence (eg, central computer/telephone; sequentially numbered, opaque, sealed containers), describing any steps to conceal the sequence until interventions were assigned | Page 5 (sealed envelopes) |
|  | Implementation | 11 | Whether the personnel who enrolled and those who assigned participants to the interventions had access to the random allocation sequence | Page 5( an independent researcher) |
|  | Blinding | 12a | Who was blinded after assignment to interventions (eg, participants, care providers, outcome assessors, data analysts) | Page 9  **Reported on page no.** |
|  |  | 12b | If blinded, how blinding was achieved and description of the similarity of interventions | Page 9 |
|  | Statistical methods | 13a | Statistical methods used to compare groups for primary and secondary outcomes, including harms | Page 9 |
|  |  | 13b | Definition of who is included in each analysis (eg, all randomised participants), and in which group | Page 9 |
|  |  |  |  |  |
|  |  | 13c | How missing data were handled in the analysis | Page 10-11 |
|  |  | 13d | Methods for any additional analyses (eg, subgroup and sensitivity analyses), distinguishing prespecified from post hoc | Page 10-11 |
|  | **Results** | | |  |
|  | Participant flow, including flow diagram | 14a | For each group, the numbers of participants who were randomly assigned, received intended intervention, and were analysed for the primary outcome | Page 12 |
|  |  | 14b | For each group, losses and exclusions after randomisation, together with reasons | Page 12(no dropouts) |
|  | Recruitment | 15a | Dates defining the periods of recruitment and follow-up for outcomes of benefits and harms | Page 5 |
|  |  | 15b | If relevant, why the trial ended or was stopped | Page 5 |
|  | Baseline data | 16 | A table showing baseline demographic and clinical characteristics for each group | Page 12 Results section |
|  | Numbers analysed,  outcomes and estimation | 17 | For each primary and secondary outcome, by group:  ● the number of participants included in the analysis  ● the number of participants with available data at the outcome time point  ● result for each group, and the estimated effect size and its precision (such as 95% confidence interval)  ● for binary outcomes, presentation of both absolute and relative effect size | Page 13-16 |
|  | Harms | 18 | All harms or unintended events in each group | None observed |
|  | Ancillary analyses | 19 | Any other analyses performed, including subgroup and sensitivity analyses, distinguishing pre-specified from post hoc | Page11  Questionnaire Data Analysis |
|  | **Discussion** | | |  |
|  | Interpretation | 20 | Interpretation consistent with results, balancing benefits and harms, and considering other relevant evidence | Page 16-18 |
|  | Limitations | 21 | Trial limitations, addressing sources of potential bias, imprecision, generalisability, and, if relevant, multiplicity of analyses | Page 19-22 |
|  |  |  |  |  |
